# Routine Physical Therapy with and without Neural Mobilization in Chronic Musculoskeletal Neck Disorders with Nerve-Related Symptoms: Systematic Review and Meta-Analysis

**DOI:** 10.3390/healthcare12121225

**Published:** 2024-06-19

**Authors:** María José López-Pardo, Andrés Calvache-Mateo, Javier Martín-Núñez, Alejandro Heredia-Ciuró, Laura López-López, Marie Carmen Valenza, Irene Cabrera-Martos

**Affiliations:** Department of Physiotherapy, Faculty of Health Sciences, University of Granada, 60 Av. De la Ilustración, 18016 Granada, Spain; lll92hs@correo.ugr.es (M.J.L.-P.); andrescalvache@ugr.es (A.C.-M.); javimn@ugr.es (J.M.-N.); ahc@ugr.es (A.H.-C.); cvalenza@ugr.es (M.C.V.); irenecm@ugr.es (I.C.-M.)

**Keywords:** neck disorders, physical therapy, neural mobilization, neuropathy

## Abstract

No previous study has evaluated the effectiveness of routine physical therapy with and without neural mobilization for patients with chronic musculoskeletal neck disorders and cervical radiculopathy. The objective is to evaluate the effectiveness of routine physical therapy with and without neural mobilization on pain and mobility in patients with chronic musculoskeletal neck disorders and cervical radiculopathy. A systematic review with meta-analysis of randomized clinical trials involving the use of neural mobilization techniques for the treatment of chronic musculoskeletal neck disorders and cervical radiculopathy was conducted. Methodological quality was assessed by the Cochrane Risk of Bias Tool and PEDro scale. Data were pooled and a meta-analysis was performed using a random effects model with Review Manager 5 software. Seven articles were included in our review. Significant differences were found in mobility but not in pain in favor of using routine physical therapy with neural mobilization for the treatment of chronic musculoskeletal neck disorders and cervical radiculopathy. Our results show that routine physical therapy accompanied by neural mobilization is superior for improving mobility in comparison with routine physical therapy alone in patients with musculoskeletal neck disorders and cervical radiculopathy.

## 1. Introduction

Neck pain is one of the most common and disabling musculoskeletal disorders, causing remarkable economic and social burden. This pain is expected to be present in up to 71% of people during their lifetime [1]. In addition, the proportion of people at risk of neck pain is expected to increase in the next decade [2].

Cervical radiculopathy is frequently associated with neck pain and includes tingling, numbness, or discomfort in the arm, upper back, and upper chest with or without an associated headache [3]. These symptoms can be extended to the neck muscles, zygapophyseal joints, intervertebral discs, nerve roots, or trunk. Daffner et al. [4] reported that neck pain spreading down the arm is more frequent than neck pain alone. In addition, higher levels of self-reported disability are reported among patients with neck pain and cervical radiculopathy [4].

Despite the severity of this condition, the choice of treatment remains controversial. Pharmacological treatment seems to be the go-to treatment [5], but conservative management is also widely used among patients with neck pain and cervical radiculopathy. Routine physical therapy is highly recommended, including electrotherapeutic modalities, neck strengthening exercises, traction, and methods for handling soft tissue [6]. However, a recent review reported that further research to facilitate practice guidelines is needed [6].

A previous review that looked at the effectiveness of neural mobilization for neuromusculoskeletal conditions of the upper and lower limbs concluded that this approach is effective in the management of nerve-related neck and arm pain, with a 1a level of evidence [7]. However, this review does not focus solely on cervical radiculopathy and calls for the need to assess function in future studies.

This technique uses the gliding of the nervous system through its anatomical pathway with postural changes, manual therapy, or exercises that involve joint movement. Different studies show that neural mobilization reduces intraneural edema and improves intraneural fluid dispersion following a nerve injury [8,9]. The goal of this technique is to restore the dynamic balance between the neural tissue and its surroundings, promoting optimal function and reducing pressure over the neural structures [9]. However, Coppieters et al. affirmed that neural mobilization not only loads the nervous system but also challenges non-neural structures [10,11,12], which has ignited an ongoing debate in the literature about the involvement of non-neural structures in the neural mobilization techniques and the implication of these structures in the treatment outcomes and testing [13,14].

In this way, neurodynamic testing and neural mobilization techniques have been widely used in multidimensional treatments for improving neural and non-neural tissue function [8,9]. A previous review showed the effectiveness of this type of technique in low back pain [13]. Likewise, the review by Su et al. [14] showed how neural tissue treatment generates significant improvements in pain and function; however, techniques other than neurodynamics were included.

The systematic review by Varangot-Reille et al. (2022) [15] observed significant improvements following neurodynamic treatment in patients with cervical pain and related nerve symptoms; however, they included patients at different stages of clinical progression. Thus, our objective is not only to evaluate the effectiveness of neurodynamics in these patients but also to compare whether adding neurodynamics to a physiotherapy treatment is more effective than physiotherapy treatment alone. No previous study has evaluated the effectiveness of routine physical therapy with and without neural mobilization for patients with chronic musculoskeletal neck disorders and cervical radiculopathy. Therefore, the objective of this systematic review with meta-analysis was to evaluate the effectiveness of routine physical therapy with and without neural mobilization on pain and mobility in this population.

## 2. Methods

### 2.1. Study Selection and Split

This review was written according to the Preferred Reporting Items for Systematic Reviews and Meta-Analysis (PRISMA) Statement [16]. We registered the review at the International Prospective Register of Systematic Reviews (PROSPERO) with registration number CRD42022380071.

An electronic search was conducted using four electronic databases: PubMed, Web of Science, Scopus, and Google Scholar. The keywords used to develop the search are shown in Table 1.

The terms used can be seen in Appendix A—Search strategy. Randomized control trial (RCT) publications were included from inception until June 2024.

To define the research question, the PICOS (participants, interventions, comparisons, outcome, and study design) model was applied. We included (1) participants with chronic musculoskeletal neck disorders with cervical radiculopathy (>3 months) [17] (2) who had received routine physical therapy with neural mobilization. (3) This intervention had to be compared to a routine physical therapy intervention alone. (4) The main outcome measures were pain and mobility. (5) Only randomized clinical trials were included.

We excluded articles that deal with subjects unrelated to this topic, articles that included patients with acute or subacute condition, those that were not available in full text, reviews, meta-analyses, observational studies, books, notes, conference proceedings, theses or dissertations, letters, and abstracts. Additionally, we screened the reference lists of relevant reviews related to the term and considered non-English-language studies for inclusion if the translation was possible. When the full text of the studies was not available either in the databases or in the repository of our university, the authors were contacted.

### 2.2. Data Extraction

After obtaining records from all the databases, duplicates were removed using Mendeley. Two reviewers (MJLP and ACM) independently assessed the titles and abstracts of all the RCTs and examined the studies to confirm their eligibility. Any conflicts were resolved by a third reviewer (JMN). Following this, each of the chosen articles was thoroughly analyzed in full text, and any disagreements were once more resolved by the third reviewer.

Data extraction was conducted following the data extraction checklist outlined in the *Cochrane Handbook for Systematic Reviews* [18]. The extracted data encompassed authors, year of publication, study design, number of patients, sex distribution, mean age, intervention description, study frequency and duration, and reported outcomes.

### 2.3. Quality Assessment

Data from the selected articles were extracted and the risk of bias of these was assessed using the Cochrane Risk of Bias Tool for Randomized Controlled Trials 2.0 [19]. This tool includes five domains, which are (1) bias arising from the randomization process; (2) bias due to deviations from the intended interventions; (3) bias due to missing outcome data; (4) bias in the measurement of the outcome; and (5) bias in the selection of the reported result. According to the recommendations, we considered a study as having a low risk of bias when judged to be at low risk of bias for all domains, and as having a high risk of bias when a trial was considered to be at risk of bias in one domain or have “some concerns” in multiple domains (three or more). If a study was judged to raise some concerns in at least one domain, but not to be at high risk of bias for any one domain, we considered it as having some concerns. In the same way as for the study selection, a risk of bias assessment was carried out independently by two reviewers and any disagreement was resolved with a third reviewer.

In addition, methodological quality was assessed using the Physiotherapy Evidence Database (PEDro) scale [20]. It consists of 11 items that assess external validity (item 1), internal validity (items 2–9), and applicability or generalizability (items 10–11). Each criterion that is clearly met receives one point, with a maximum possible score of 11 points, indicating the highest methodological quality for a randomized controlled trial.

### 2.4. Data Synthesis and Analysis

Review Manager 5 software (5 (Rev-Man version 5.1, updated March 2011) was used to perform a meta-analysis in all RCTs that included pain and mobility. The sample size, means, standard mean differences, and standard deviations (SDs) to the post-intervention for each variable were introduced in the software program. When means and standard deviations for the results were not provided, the authors were contacted. Ultimately, variables expressed in non-comparable units were excluded from the meta-analysis.

Where standard deviations were missing, but *p*-values or 95% confidence intervals were given, these were calculated via the embedded Review Manager calculator. If studies used different measuring tools, the chosen measure of effect size was the standard mean difference (SMD).

The overall mean effect sizes were estimated using random effect models according to statistical heterogeneity I^2^ tests and expressed effects as mean differences (MDs) and standard mean differences (SMDs) with accompanying confidence intervals. Visual inspection of the forest plots for outlier studies was also undertaken. I^2^ describes the percentage of total variation across studies that is due to heterogeneity rather than chance [21].

## 3. Results

**Figure 1:**
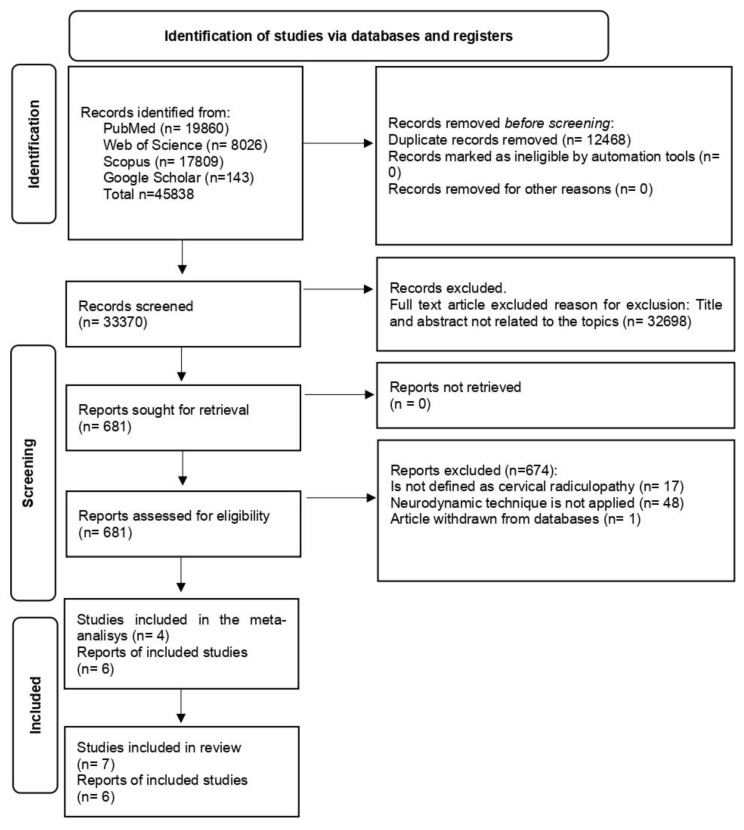
PRISMA flowchart.

A total of 45,838 studies was identified. After removed the duplicates, a total of 33,370 articles was identified. Additionally, articles were excluded based on their titles and abstracts (n = 32,698), as well as those not meeting the inclusion and exclusion criteria (n = 674). As a result, a total of seven studies [22,23,24,25,26,27,28] were included in this systematic review, of which four were included in the meta-analysis. The search results and final included studies are shown in Figure 1.

The characteristics of the distribution, demographics, and profile of the participants and the methodological quality of the studies are shown in Table 2.

A total of 285 patients with chronic neck pain and cervical radiculopathy were recruited. The age of the patients ranged from 29 to 50. Regarding the presence of nerve-related symptoms, the seven studies included in this review considered the pain provocation test as a criterion for inclusion.

Descriptions of the interventions, variables, and results obtained can be found in Table 3.

The most frequently used variables were the NPRS and VAS for assessing pain, and cervical range of motion (ROM) for mobility.

The number of routine physical therapy sessions ranged between 6 and 24, being applied over a period of 3 to 9 weeks. However, the intervention in the Marks et al. (2011) [27] study consisted of a single session of neurodynamics for the experimental group and accessory and passive cervical and rib mobilization for the control group.

In relation to the control group intervention, all received routine physical therapy with a duration, frequency, and number of sessions identical to the experimental group. The most commonly used technique in this standard physical therapy intervention was cervical traction [22,23,24,26], used in four of the seven included studies; other techniques used were home exercise, relaxation and self-help strategies [25,28], manual therapy such as accessory and passive cervical and rib mobilization [27], and electrotherapy [23,24].

None of the included studies conducted patient follow-up. The majority of the results were evaluated immediately after the intervention, only two studies carried out a one-week follow-up [27] and 52-week follow up [25], finding the same results as after the intervention in both cases.

**Figure 2:**
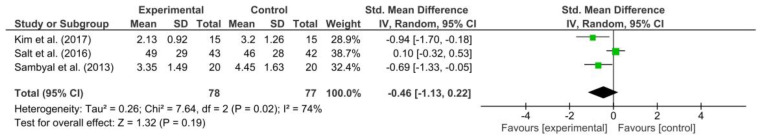
Forest plot of the meta-analysis for the pain [24,25,26].

The results of the meta-analysis for the pain are included in Figure 2. Routine physical therapy with neural mobilization shows no significant improvements compared to routine physical therapy without neural mobilization (SMD) = −0.46; 95% CI = −1.13; 0.22; *p* = 0.19.

**Figure 3:**
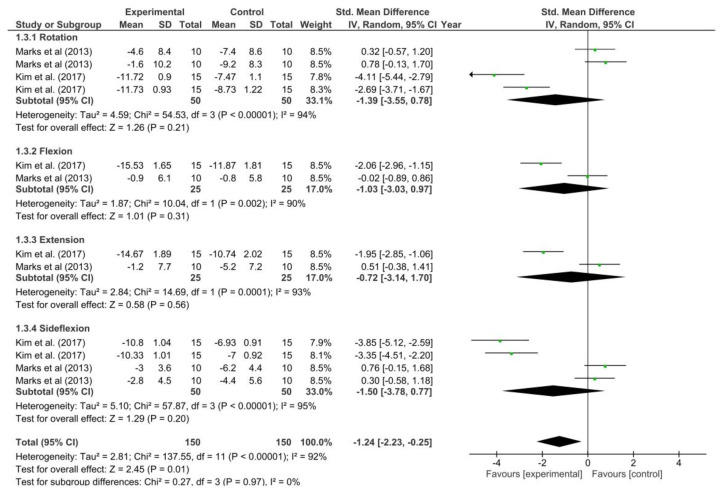
Forest plot of the meta-analysis for the Neck Disability Index [24,27].

The forest plot including mobility is shown in Figure 3. The results showed significant improvements with the use of routine physical therapy with neural mobilization compared to routine physical therapy without neural mobilization (SMD = −1.24; 95% CI = −2.23; −0.25; *p* = 0.01).

**Figure 4:**
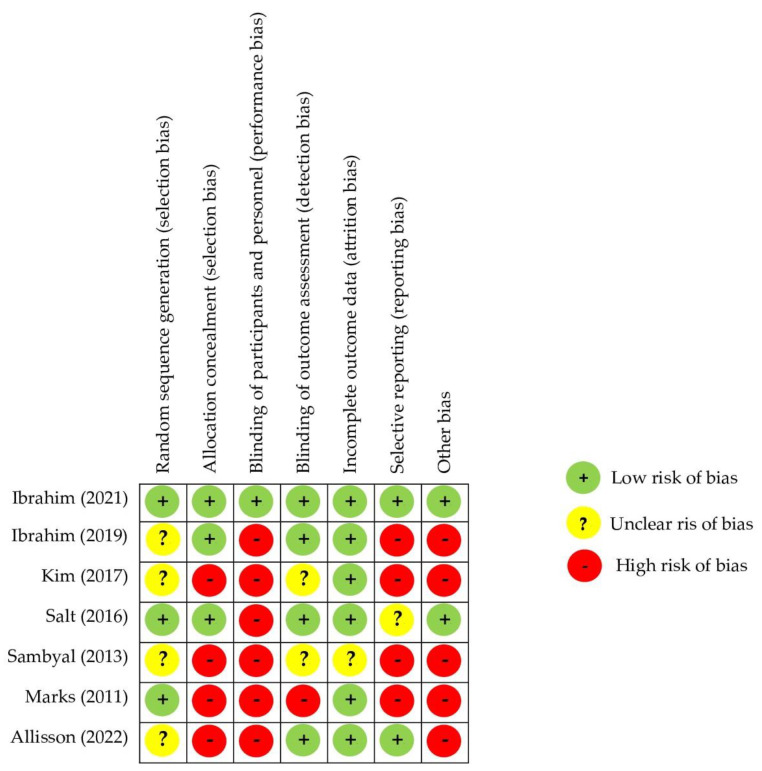
Risk of bias summary [22,23,24,25,26,27,28].

Figure 4 shows the RoB 2 summary; 57,14% of the studies had at least three domains with “low risk”, while 71.42% had two or more domains with “high risk”.

The methodological quality assessment by PEDro scale revealed high quality across the studies included in this systematic review, except for the study of Sambyal et al. (2013) [26], which obtained three points. The average PEDro scale score was 5.57 points out of 11 (Table 4).

## 4. Discussion

This review aimed to evaluate the efficacy of routine physical therapy with and without neural mobilization on pain and mobility in patients with chronic musculoskeletal neck disorders with cervical radiculopathy. The results show that routine physical therapy with neural mobilization is superior for improving mobility but not pain in this population.

Previous studies have explored the effects of neural mobilization in patients with pain. A systematic review carried out in 2017 by Basson et al. [7] revealed that there were benefits from the use of neural mobilization for neck and back pain but did not find clear answers for their effects on other musculoskeletal conditions. Another systematic review conducted by Tiago et al. reported benefits from the use of neural mobilization, either combined with other treatments (e.g., exercise) or used in isolation, being superior to control interventions for the treatment of low back pain [13]. These results were in line with the ones described by Ellis and Hing et al. [9] in a different systematic review that examined the therapeutic efficacy of neural mobilization. In this review, eight out of the eleven studies included showed neural mobilization had positive effects on pain and disability [9]. Su et al. synthesized the evidence for neural tissue management effectiveness in participants with nerve-related chronic musculoskeletal pain. The results from this review suggested that neural mobilization was superior to minimal intervention for the reduction of pain and disability in individuals with nerve-related chronic musculoskeletal pain [14]. In our systematic review, the meta-analysis results were in favor of routine physical therapy with neural mobilization compared to routine physical therapy without neural mobilization.

Some of the benefits of neural mobilization techniques include an improvement in tissue mobility, circulation to the nerves, and axonal transport, key for the structure and function of nerves [29]. Sharaf et al. [30] also found that routine physical therapy was superior to routine physical therapy without neural mobilization pain, functional disability, and H-reflex in patients who underwent decompressive laminectomy. The explanations reported included the repercussions of neural mobilization on the dissipation of the edema, alleviation of the hypoxia, a reduction in symptoms related to the compression of the nerves, and an improvement in nerve conduction [31]. In this line, the randomized controlled trial conducted by Kayiran and Turhan [32] in patients with cervical disc herniation also showed that routine physical therapy with neural mobilization allows for better results in cervical posture, pain, and active range of motion.

Our results support the use of routine physical therapy with neural mobilization as a therapeutic intervention for the treatment of chronic neck pain and cervical radiculopathy. Nonetheless, our systematic analysis of the literature must be interpreted with caution due to the difference in dosages, timings, and different techniques of interventions found among the studies. We must also take into account the risk of bias when interpreting the results, attributing this risk of bias mainly to the inadequate blinding of the participants to their intervention group.

### Study Limitations

Despite the review of multiple electronic databases, it could be possible to have missed some trials. In addition, the heterogeneity of neural mobilization interventions used in the different randomized controlled trials makes the treatments too heterogeneous to make firm conclusions. The majority of studies had low numbers of participants and the age ranged from 29 to 50 years; therefore, the results are not necessarily transferable to all the population and the severity of the symptoms was not taken into consideration in any of the included articles. Only three articles specified the chronicity of the symptoms.

Future studies should be more precise with the interventions both in terms of homogeneity and in providing details on the progression of these interventions. It would also be interesting to analyze other variables such as strength or range of motion, as they are potential for improvement. Finally, the absence of medium- or long-term follow-up precludes understanding the effectiveness of these treatments over time. Future studies should include patient follow-up to determine the duration of the observed improvements.

## 5. Conclusions

The use of routine physical therapy accompanied by neural mobilization is more effective than routine physical therapy without neural mobilization to improve mobility but not pain in patients with chronic neck pain and cervical radiculopathy. Our results show that neural mobilization can be considered a useful tool in pain management and functionality improvement in this group of patients. The findings of this review make a significant contribution to clinical practice regarding the management of neck pain with nerve-related symptoms.

## Figures and Tables

**Table 1:** Keywords used for the search strategy.

Population	Intervention	Control	Design
Radiculopathy Musculoskeletal pain Referred pain Nerve tissue/injuries Radicular pain Nerve pain Neuropathy Compressive neuropathy Nerve entrapment Entrapment neuropathies Nerve compression Neural compression Neck Pain Neck pain * Cervical pain * Cervical spine pain Cervicalgia * Cervicodynia	Nerve tissue/therapy Nerve treatment Neurodynamic * Nerve stretch * Neural treatment Nerve tensión Neural tensión Nerve Slide Nerve mobili * Neural mobili * Nerve glid * Neural glid *	Conservative intervention Physical approach Physical intervention Physical management Physical therapy Physiotherapy Manual therapy	Randomized controlled trial Clinical trial Randomised control * Randomized control * Randomised control trial Randomized control trial Controlled clinical trial Randomi *

**Table 2:** Study design, participant profile, and participant distribution.

Author (Year)	Study Design	Participant Distribution	Gender (% Female)	Mean Age (SD)	Clinical’s Profile Criteria
Ibrahim (2021) [22]	RCT	EG (n = 20) CG (n = 20)	NR	NR	>3 criteria of Wainner
Ibrahim (2019) [23]	RCT	EG (n = 20) CG (n = 20)	EG: NR CG: NR	EG: NR CG: NR	>3 criteria of Wainner
Kim (2017) [24]	RCT	EG (n = 15) CG (n = 15)	EG: 60 CG: 66.6	EG: 29.27 (3.34) CG: 29.22 (3.07)	>3 criteria of Wainner and limited range of movement of the upper limb.
Salt (2016) [25]	RCT	EG (n = 43) CG (n = 42)	EG: 47 CG: 53	EG: 47 (11) CG: 47 (11)	Patients’ subjective presentation of the pain pattern. It therefore included patients with somatic referred pain and neurogenic radiating symptoms.
Sambyal (2013) [26]	RCT	EG: (n = 20) CG (n = 20)	EG: NR CG: NR	EG: NR CG: NR	>3 criteria of Wainner
Marks (2011) [27]	RCT	EG: (n = 10) CG: (n = 10)	EG: 80 CG: 80	EG: 52.6 (12.5) CG: 53.7 (9.0)	Neck pain radiating into upper extremity with limited cervical ROM and a positive ULNT 1.
Allisson (2022) [28]	RCT	EG: (n = 10) CG1: (n = 10) CG2: (n = 10)	EG: 60 CG1: 80 CG2: 60	EG: NR CG1: NR CG2: NR	Definitions of cervicobrachial pain syndrome as proposed by Elvey and Hall (1997).

SD: standard deviation; RCT: randomized controlled trial; EG: experimental group; CG: control group; NR: not reported; ULNT: upper limb neurodynamic test.

**Table 3:** Intervention’s description, main outcomes, and results obtained.

Author (Year)	Experimental Intervention	Duration/Frequency/Number of Sessions of EG	Control Intervention	Duration/Frequency/Number of Sessions of CG	Main Outcomes	Main Results
Ibrahim (2021) [22]	NM combined with RPT	9 weeks/3 times × week/ 12 sessions	RPT (manual traction and infrared irradiation)	9 weeks/3 times × week/ 12 sessions	Pain: VAS Mechanosensitivity: ULTT-1	No significant difference between groups regarding both pain and mechanosensitivity.
Ibrahim 2019 [23]	NM combined with RPT	3 weeks/3 times × week/9 sessions	RPT (CT + FS + IR)	3 weeks/3 times × week/9 sessions	Pain: VAS Grip strength: dynamometry	No significant difference between groups regarding both pain and grip strength.
Kim (2017) [24]	NM combined with RPT	8 weeks/3 times × week/24 sessions	RPT (CT + TENS)	8 weeks/3 times × week/24 sessions	Pain: NPRS Function: NDI cervical mobility: ROM deep flexor endurance: CCFT	Significant differences between groups at 8 weeks in pain, function, cervical mobility, and deep flexor endurance.
Salt (2016) [25]	NM + SM	6 weeks/1 time/week/6 sessions	SM (home exercise, relaxation, and self-help strategies)	6 weeks/1 time/week/6 sessions	Pain intensity: VAS Symptoms: GROG Function: NULI	No statistically significant between-group differences were found at the end of the intervention or at 52 week follow-up.
Sambyal (2013) [26]	NM + CT	4 weeks/4 times per week/16 sessions	RPT (CT)	4 weeks/4 times per week/16 sessions	Pain intensity: VAS	NM group improved significantly more in pain intensity (*p* < 0.05) than control group.
Marks (2011) [27]	NM	1 session	RPT (accessory and passive cervical and rib mobilization)	1 session	Pain intensity: VAS CROM: flexion, extension, rotation, and latero-flexion Mechanosensitivity: elbow angle	No significant differences were found between groups after the intervention or at 1 week follow-up (*p* > 0.05).
Allisson (2022) [28]	NM	8 weeks	CG1: RPT (mobilization, stretching, and exercise) CG2: no treatment	8 weeks	Subjective pain experience: SF-MPQ Pain intensity: VAS and NPQ	NM group and articular group had significant improvement in all variables. No differences between NM and articular groups for the subjective pain experience and neck pain; however, NM had significantly lower pain intensity than the articular group at the end of the treatment (*p* < 0.05).

NM: neural mobilization; RPT: routine physical therapy treatment; VAS: visual analogue scale; ULTT-1: Upper Limb Tension Test-1; CT: cervical traction; FS: flexion stretching; IR: infrared radiation; NPRS: Numeric Pain Rating Scale; ROM: range of motion; TENS: transcutaneal electrical nerve stimulation; NDI: Neck Disability Index; CCFT: Cranio-Cervical Flexion Test; SM: self-management; GROC: Global Rating of Change; NULI: Neck and Upper Limb Index; SF-MPQ: Short-form Mcgill Pain Questionnaire; NPQ: Neck Pain Questionnaire.

**Table 4:** Assessment of the studies’ methodological quality based on the PEDro scale.

Authors	Items											
	1	2	3	4	5	6	7	8	9	10	11	Total
Ibrahim (2021) [22]	1	1	1	1	0	0	0	1	0	1	1	6
Ibrahim (2019) [23]	1	1	0	1	0	0	1	1	1	1	0	6
Kim (2017) [24]	1	1	0	1	0	0	0	1	1	1	1	6
Salt (2016) [25]	1	1	1	1	0	0	1	0	1	1	1	7
Sambyal (2013) [26]	1	1	0	1	0	0	0	0	0	1	0	3
Marks (2011) [27]	1	1	0	1	0	0	0	1	1	0	1	5
Allisson (2022) [28]	1	1	0	0	0	0	1	1	1	1	1	6

## Data Availability

Data are contained within the article.

## Appendix A. Search Strategy

SCOPUS: ((“Nerve tissue/therapy” OR “Nerve treatment” OR “Neural treatment” OR neurodynamic* OR “Nerve stretch*” OR “Nerve tension” OR “Neural tension” OR “Nerve Slide” OR “Nerve mobili*” OR “Neural mobili*” OR “Nerve glid*” OR “Neural glid*” OR “Conservative intervention” OR “Conservative approach” OR “Conservative management” OR “Conservative therap*” OR “Physical approach” OR “Physical intervention” OR “Physical management” OR “Physical therapy” OR physiotherapy OR “Manual therapy”) AND (“Neck Pain”[Mesh] OR “neck pain*” OR “cervical pain*” OR “cervical spine pain” OR “cervicalgia*” OR “cervicodynia*” OR radiculopathy OR “Musculoskeletal pain” OR “Referred pain” OR “Nerve tissue/injuries” OR “Radicular pain” OR “Nerve pain” OR neuropathy OR “compressive neuropathy” OR “nerve entrapment” OR “entrapment neuropathies” OR “nerve compression” OR “neural compression”) AND (“Randomized controlled trial” OR “Clinical trial” OR “Randomised control*” OR “Randomized control*” OR “Randomised control trial” OR “Randomized control trial” OR “Controlled clinical trial” OR “Randomi*” OR rct OR trial OR placebo OR group*)) AND (LIMIT-TO ( DOCTYPE, “ar”)) AND (LIMIT-TO (EXACTKEYWORD, “Human”) OR LIMIT-TO (EXACTKEYWORD, “Humans”) OR LIMIT-TO (EXACTKEYWORD, “Adult” )).

WEB OF SCIENCE: TS = ((“Nerve tissue/therapy” OR “Nerve treatment” OR “Neural treatment” OR Neurodynamic* OR “Nerve stretch*” OR “Nerve tension” OR “Neural tension” OR “Nerve Slide” OR “Nerve mobili*” OR “Neural mobili*” OR “Nerve glid*” OR “Neural glid*” OR “Conservative intervention” OR “Conservative approach” OR “Conservative management” OR “Conservative therap*” OR “Physical approach” OR “Physical intervention” OR “Physical management” OR “Physical therapy” OR Physiotherapy OR “Manual therapy”) AND (“Neck Pain”[Mesh] OR “neck pain*” OR “cervical pain*” OR “cervical spine pain” OR “cervicalgia*” OR “cervicodynia*” OR Radiculopathy OR “Musculoskeletal pain” OR “Referred pain” OR “Nerve tissue/injuries” OR “Radicular pain” OR “Nerve pain” OR Neuropathy OR “compressive neuropathy” OR “nerve entrapment” OR “entrapment neuropathies” OR “nerve compression” OR “neural compression”) AND (“Randomized controlled trial” OR “Clinical trial” OR “Randomised control*” OR “Randomized control*” OR “Randomised control trial” OR “Randomized control trial” OR “Controlled clinical trial” OR “Randomi*” OR RCT OR Trial OR Placebo OR Group*)).

PUBMED: (“Nerve tissue/therapy” OR “Nerve treatment” OR “Neural treatment” OR Neurodynamic* OR “Nerve stretch*” OR “Nerve tension” OR “Neural tension” OR “Nerve Slide” OR “Nerve mobili*” OR “Neural mobili*” OR “Nerve glid*” OR “Neural glid*” OR “Conservative intervention” OR “Conservative approach” OR “Conservative management” OR “Conservative therap*” OR “Physical approach” OR “Physical intervention” OR “Physical management” OR “Physical therapy” OR Physiotherapy OR “Manual therapy”) AND (“Neck Pain”[Mesh] OR “neck pain*” OR “cervical pain*” OR “cervical spine pain” OR “cervicalgia*” OR “cervicodynia*” OR Radiculopathy OR “Musculoskeletal pain” OR “Referred pain” OR “Nerve tissue/injuries” OR “Radicular pain” OR “Nerve pain” OR Neuropathy OR “compressive neuropathy” OR “nerve entrapment” OR “entrapment neuropathies” OR “nerve compression” OR “neural compression”)

GOOGLE SCHOLAR: (“Nerve tissue/therapy” OR “Nerve treatment” OR “Neural treatment” OR Neurodynamic* OR “Nerve stretch*” OR “Nerve tension” OR “Neural tension” OR “Nerve Slide” OR “Nerve mobili*” OR “Neural mobili*” OR “Nerve glid*” OR “Neural glid*” OR “Conservative intervention” OR “Conservative approach” OR “Conservative management” OR “Conservative therap*” OR “Physical approach” OR “Physical intervention” OR “Physical management” OR “Physical therapy” OR Physiotherapy OR “Manual therapy”) AND (“Neck Pain”[Mesh] OR “neck pain*” OR “cervical pain*” OR “cervical spine pain” OR “cervicalgia*” OR “cervicodynia*” OR Radiculopathy OR “Musculoskeletal pain” OR “Referred pain” OR “Nerve tissue/injuries” OR “Radicular pain” OR “Nerve pain” OR Neuropathy OR “compressive neuropathy” OR “nerve entrapment” OR “entrapment neuropathies” OR “nerve compression” OR “neural compression”).
